# A Case of Mixed Connective Tissue Disease That Transformed Into Systemic Lupus Erythematosus After a Long Clinical Course

**DOI:** 10.7759/cureus.38201

**Published:** 2023-04-27

**Authors:** Fumikazu Sato, Momoka Sato, Takahiro Yamano, Kaori Yamaguchi, Taito Miyake

**Affiliations:** 1 Division of Nephrology and Rheumatology, Kouseiren Takaoka Hospital, Takaoka, JPN

**Keywords:** tacrolimus, autoantibody, lupus nephritis, systemic lupus erythematosus, mixed connective tissue disease

## Abstract

Mixed connective tissue disease (MCTD), a multisystem autoimmune disease that was first proposed in 1972, has overlapping features with other autoimmune diseases. In recent studies, mixed connective tissue disease has been reported to change into other connective tissue diseases (CTD; such as systemic lupus erythematosus [SLE], polymyositis, and systemic sclerosis [SSc]) in the long term. We report the case of a 58-year-old Japanese man diagnosed with mixed connective tissue disease 15 years ago. During his clinical course, he developed discoid lupus erythematosus, pancytopenia, a low complement titer, proteinuria, and hematuria. He also turned positive for the anti-double-stranded deoxyribonucleic acid (dsDNA) antibody. A kidney biopsy revealed lupus nephritis (LN) class IV. Therefore, we considered this to be a shift from mixed connective tissue disease to systemic lupus erythematosus. We changed his treatment to lupus nephritis, after which he remained in remission. Our case suggests that mixed connective tissue disease may shift to other connective tissue diseases over a long period; therefore, it is necessary to identify whether patients with mixed connective tissue disease fulfill the diagnostic criteria for other connective tissue diseases when new manifestations appear.

## Introduction

Mixed connective tissue disease (MCTD) is a multisystem disease with overlapping features of other autoimmune diseases, including systemic lupus erythematosus (SLE), polymyositis, and systemic sclerosis (SSc), along with the presence of anti-U1-ribonucleoprotein (RNP) antibodies [1]. MCTD often responds well to steroids and has a good prognosis. MCTD was first proposed in 1972; however, there is still a lack of data on its long-term course. In recent years, some studies have reported that patients with MCTD sometimes fulfill the diagnostic criteria of another connective tissue disease (CTD) and that their diagnosis changes during their clinical course [2,3]. However, reports on the clinical profile conversion from MCTD to another CTD are relatively rare.

SLE, another multisystem autoimmune disease characterized by the production of autoantibodies, often involves the kidneys [4]. Lupus nephritis (LN) is a common renal manifestation that can lead to renal failure in patients with SLE. LN is classified into six histological types according to the 2003 International Society of Nephrology/Renal Pathology Society (ISN/RPS) classification [5]. Patients with proliferative forms of LN (class Ⅲ, Ⅳ, Ⅲ/Ⅳ, and Ⅴ) are at high risk of developing end-stage kidney disease. Hence, it is crucial for patients with SLE to receive appropriate treatment to maintain long-term kidney function [6].

Here, we describe a case of LN during a follow-up of MCTD diagnosed 15 years ago, which we successfully managed with an SLE-based therapeutic approach. We then discuss the long-term conversion of MCTD to other autoimmune diseases.

## Case presentation

A 58-year-old Japanese man was diagnosed with MCTD 15 years prior based on the presence of swollen fingers, positive anti-RNP antibodies, inflammatory myopathy, and finger skin sclerosis. Five years ago, he had a flare-up of inflammatory myopathy with leukopenia, thrombocytopenia, and scattered erythema multiforme. His symptoms did not meet the 1997 American College of Rheumatology (ACR) revised criteria for the classification of SLE [7]. He was diagnosed with a relapse of MCTD and was administered an increased dose of steroids, which improved his symptoms. Thereafter, he was treated with a maintenance dose of prednisolone (PSL) of 5 mg/day for five years.

He then presented to our department with arthritis and skin symptoms. On physical examination, his temperature was 36.5 °C, his heart rate was 65 beats per minute, his blood pressure was 156/72 mmHg, his respiratory rate was 16 breaths per minute, and his oxygen saturation was 92% while breathing ambient air. Fine crackling sounds were heard in the lower lung. His heart rhythm was regular and without cardiac murmurs. His abdomen was soft, non-tender, and non-distended, with normal bowel sounds and no appreciable organomegaly. The patient had hyperpigmented patches on his face and fingers, which were consistent with discoid lupus erythematosus. There was no edema in the legs or feet. His joints were non-tender, without swelling. His grip strength dropped from 28/32 kg to 13/12 kg because of arthralgia, but all other motor and sensory functions were normal.

Laboratory findings (Tables 1-2) showed leukopenia, thrombocytopenia, a low complement titer, microhematuria, and proteinuria. His serum creatinine and CRP levels were normal. Urinalysis showed proteinuria (protein-creatinine ratio: 1.3 g/gCr), dysmorphic red blood cells (RBC) 10-19/high power field, and RBC casts 1-4/low power field. The electrocardiogram findings were normal. Chest computed tomography showed subpleural, symmetrical, bilateral ground-glass opacities with traction bronchiectasis. His serum anti-nuclear antibody (ANA) titer was 1/1280 and positive for anti-RNP and anti-Ro (SS-A) antibodies. The anti-double-stranded deoxyribonucleic acid (dsDNA) antibody was > 380 U/mL, but the anti-Smith (SM) antibody was negative. Table 2 summarizes the immune laboratory tests conducted 15 years prior, 5 years prior, and at this admission. The patient fulfilled the American Rheumatological Association criteria for SLE and received a definitive diagnosis of SLE.

**Table 1:** Laboratory findings

Laboratory test	Unit	Patient’s laboratory values	Normal range	Laboratory test	Unit	Patient’s laboratory values	Normal range
White blood cell	/mL	1900	3200–7900	HbA1c		5.9%	4.6–6.2%
Hemoglobin	g/dL	12.8	11.3–15.0	Blood glucose	g/dL	113	73–109
Hematocrit		37.3%	34.0–46.3%	IgG	mg/dL	1070	870–1700
Platelet	10^4^/mL	14.4	15.5–35.0	ANA	SP	1280	<40
Total protein	g/dL	6.0	6.9–8.4	Anti-SM	U/mL	5.7	<7.0
Albumin	g/dL	3.3	3.9–5.2	Anti-SS-B	U/mL	0.5	<7.0
AST	IU/L	34	13–33	Anti-Scl70	U/mL	<0.6	<7.0
ALT	IU/L	30	10–42	Anti-centromere	U/mL	<0.5	<7.0
LDH	IU/L	287	119–229	Anti-CL	U/mL	13	<10
ALP	IU/L	180	117–350	Anti-β2GPI	U/mL	<1.3	<3.5
gGTP	IU/L	38	9–109	Urinalysis			
Urea nitrogen	mg/dL	23.0	8–21	pH		6.0	
Creatinine	mg/dL	0.94	0.46–0.78	Glucose		Negative	Negative
eGFR	mL/min/1.73 m^2^	65		Protein		3+	Negative
Uremic acid	mg/dL	5.2	2.5–7.0	Blood		3+	Negative
CRP	mg/dL	0.05	<0.3	RBC	/HF	10–19	Negative
Na	mmol/L	140	139–146	WBC	/HF	20–29	Negative
K	mmol/L	4.2	3.7–4.8	RBC cast	/WF	5-9	Negative
Cl	mmol/L	104	101–109	WBC cast	/WF	1-4	Negative

**Table 2:** The time course of the immune laboratory tests Anti-RNP antibody assay system changed between 15 years prior and 5 years prior.

	15 years prior	5 years prior	Current admission	Normal range
Anti-RNP antibody	119.8 index (EIA)	8.1 U/mL (FEIA)	5.2 U/mL (FEIA)	EIA: <15 FEIA: <3.5
Anti-ds-DNA antibody (IU/mL)	No data	2.0	≧380	<10.0
Anti-sm antibody (U/mL)	No data	17.0	5.7	<7.0
Anti-SS-A antibody (U/mL)	No data	73.7	13.9	<7.0
CK (U/mL)	8427	88	106	59–248
C3 (mg/dL)	No data	58	41	73–138
C4 (mg/dL)	No data	3	4	11–31
CH50 (U/mL)	No data	15	8	30–45

**Figure 1:**
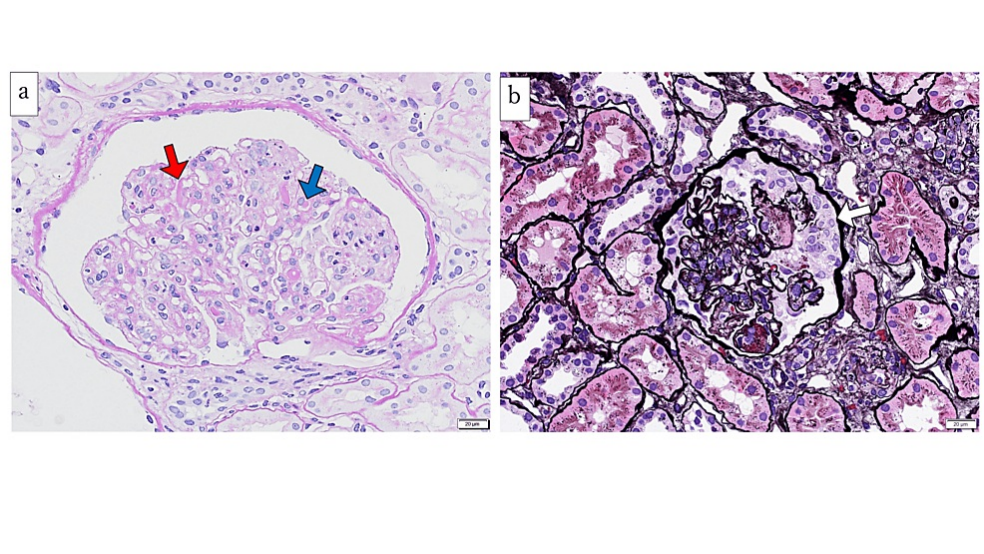
Histopathology of the renal biopsy Periodic acid-Schiff (PAS) stain (A) and periodic acid-methenamine-silver (PAM) stain (B) of light microscopy are shown (magnification × 400). PAS staining revealed endocapillary proliferation, hyaline thrombi (blue arrow), and wire-loop lesions (red arrow). PAM staining revealed cellular crescents (white arrow).

**Figure 2:**
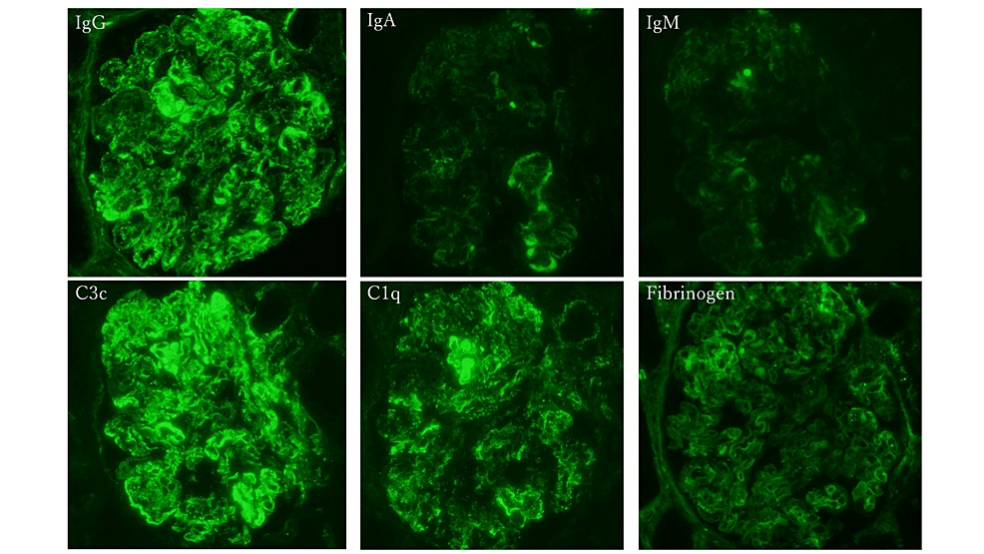
The immunofluorescence examination Immunofluorescence studies revealed a full-house pattern with mesangial and capillary wall deposits of IgG, IgA, IgM, C1q, C3c, and fibrinogen (magnification × 400).

We performed a renal biopsy to reveal the pathological findings and determine the therapeutic strategy. Light microscopy showed diffuse proliferative global glomerulonephritis with the following findings: endocapillary proliferation, hyaline thrombi, wire-loop lesions, karyorrhexis, and cellular crescents (Figure 1). On immunofluorescence examination, the biopsy specimens showed a full-house pattern with mesangial and capillary wall deposits of IgG, IgA, IgM, C1q, C3c, and fibrinogen (Figure 2). Electron microscopy showed that electron-dense deposits were located in the subepithelial space and formed spike lesions. Mesangial and subendothelial deposits were also partially detected. Consequently, we diagnosed Class IV (A) + V LN based on the ISN/RPS classification.

We considered that MCTD shifted to SLE and increased the PSL dose to 40 mg with hydroxychloroquine (HCQ) and tacrolimus initiation. His hand swelling, skin rash, proteinuria, and hematuria gradually improved. He is now on maintenance therapy with PSL 5 mg, HCQ, and tacrolimus 3 mg once daily, with complete remission of proteinuria and without any complications.

## Discussion

We diagnosed the patient with MCTD 15 years prior based on swollen fingers, positive anti-RNP antibodies, inflammatory myopathy, and finger skin sclerosis. At that time, he did not fulfill the ACR1997 criteria for the diagnosis of SLE. On this admission, the patient had a high level of anti-ds-DNA antibodies, low complement levels, pancytopenia, and pathologically proved LN (Class Ⅳ(A)+Ⅴ); the patient was diagnosed with SLE, fulfilling the EULAR/ACR 2019 criteria [8].

MCTD was defined by Sharp et al. in 1972. Recently, the number of MCTD cases has increased. Epidemiological studies showed that 84% of women (mean age, 48.1 years) in a prospective cohort study in Minnesota from 1985 to 2014 and 70% of women (mean age, 37.9 years) in a cross-sectional study in Norway were diagnosed with MCTD [9]. In Japan, 93% of patients were female (mean age, 45 years), and the estimated age of onset was 36 years. Our case involves an elderly man; in this respect, it is rare compared to recent epidemiological findings.

There are limited data on the long-term course of MCTD, but some reports suggest that MCTD may change to other CTDs (such as SLE, polymyositis, and SSc) over a long period of time. In a cohort study of long-term survivors of MCTD in Norway, 104 of 147 patients remained with their MCTD diagnosis, and 14 changed their diagnosis, of which six changed to SLE [2]. In that study, the clinical symptoms of the stable MCTD phenotype and diagnostic converters were almost identical; only the prevalence of puffy hands was higher in the stable MCTD phenotype. Another retrospective study reported that 9.1% of patients who had already been diagnosed with MCTD evolved into SLE [10]. In this report, they revealed that the presence of anti-DNA antibodies at the onset of MCTD was associated with evolution into SLE and that patients with MCTD more than five years after onset were more likely to progress to other connective tissue diseases.

MCTD is often considered a distinct disease, but it is sometimes thought to be an early manifestation of other autoimmune diseases such as SLE [11]. Differentiating MCTD and SLE is particularly important because anti-RNP antibodies, one of the diagnostic criteria for MCTD, can also appear in patients with SLE. In SLE cases, the period between the appearance of antibodies and the onset of clinical symptoms is different. However, a previous report revealed an association between disease onset and autoantibody profiles. In an American study, 115 of 130 patients who were later diagnosed with SLE had at least some autoantibodies positive for SLE before diagnosis [12]. They were divided into two groups based on the course of the disease: those who developed SLE over several years (antinuclear, anti-Ro, anti-La, antiphospholipid antibodies) and those who developed it within a few months (anti-SM, anti-RNP) after the onset of their clinical symptoms. The anti-dsDNA antibody-positive cases showed intermediate features. The anti-SM and anti-RNP antibody dual-positive cases showed a trend toward early onset, with an average age of 0.59 years. In our case, the patient had been positive for anti-RNP antibodies for 15 years and did not develop SLE for 5 years after being found to be positive for anti-SM antibodies.

When the disease concept of MCTD was first proposed, MCTD was considered not to be associated with renal impairment. However, some studies have recently reported renal involvement, which is mostly asymptomatic with a low prevalence of 0-11% [3,13]. In a cohort study of MCTD cases from 1985 to 2014, only 2 of 50 patients had glomerulonephritis during the follow-up period, both of whom were later diagnosed with SLE. Of the MCTD cases with renal involvement that did not progress to other CTDs, the main clinical manifestation was proteinuria with little haematuria. It has also been reported that the most common pathological finding is membranous nephropathy [14]. Renal involvement is infrequent, and it presents mainly as proteinuria rather than haematuria in patients with MCTD. Therefore, we concluded that the renal findings in our patient were consistent with LN.

The basic treatments for SLE are steroids and HCQ [8]. In our case, leukopenia and thrombocytopenia were observed when the patient was diagnosed with SLE. Mycophenolate mofetil (MMF) and cyclophosphamide are available for the treatment of SLE, but their side effects include pancytopenia. Tacrolimus has been reported to be as effective as MMF in treating LN [15] and maybe a better treatment option for patients with pancytopenia. Therefore, we selected tacrolimus in addition to PSL and HCQ for remission induction therapy.

## Conclusions

We successfully controlled disease activity by modifying the treatment strategy based on the new onset of SLE in the long-term course of a patient with MCTD. Because MCTD may shift to another CTD during the course of the disease, it is necessary to confirm whether other diagnostic criteria are met when new clinical findings are observed. There are still many unanswered questions about MCTD, especially regarding its long-term clinical course and predictive factors for its transformation into another CTD. If we can predict their precise clinical course, we may be able to provide more appropriate treatments. We hope that more studies will be conducted in the future to elucidate the pathogenesis of MCTD.
